# Supplementary material for: Association of ABCB1 Polymorphisms with Efficacy and Adverse Drug Reactions of Valproic Acid in Children with Epilepsy
Source: Pharmaceuticals (Basel). 2023 Oct 30;16(11):1536. doi: 10.3390/ph16111536 (PMC10675623; doi:10.3390/ph16111536)
Supplement: Supplementary file 1 [file pharmaceuticals-16-01536-s001.zip › pharmaceuticals-2622174-supplementary.pdf]

Supplementary Material

Association of *ABCB1* Polymorphisms with Efficacy and Adverse Drug Reactions of Valproic Acid in Children with Epilepsy

Jiahao Zhu [1,2], Jieluan Lu [1], Yaodong He [1,2], Xianhuan Shen [1,2], Hanbing Xia [2], Wenzhou Li [2], Jianping Zhang [1], Xiaomei Fan [2,*]

*** Correspondence:** Xiaomei Fan: xmfane@163.com

1 Supplementary Tables

Table S1 Comparison of *ABCB1* rs3789243 genotypes distribution in the persistent seizure and seizure-free groups

Genetic Model	Genotype	Persistent Seizure (*n*=61)	Seizure-Free (*n*=109)	OR (95% CI)	*p*-value
Allele contrast	C *vs.* T	78 (63.9%)	143 (65.6%)	1.00	0.758
		44 (36.1%)	75 (34.4%)	0.93 (0.59-1.48)	
Codominant	CC *vs.* CT *vs.* TT	24 (39.3%)	46 (42.2%)	1.00	0.940
		30 (49.2%)	51 (46.8%)	0.89 (0.45-1.73)	
		7 (11.5%)	12 (11.0%)	0.89 (0.31-2.57)	
Dominant	CC *vs.* CT + TT	24 (39.3%)	46 (42.2%)	1.00	0.720
		37 (60.7%)	63 (57.8%)	0.89 (0.47-1.68)	
Recessive	CC + CT *vs.* TT	54 (88.5%)	97 (89.0%)	1.00	0.930
		7 (11.5%)	12 (11.0%)	0.95 (0.35-2.57)	
Overdominant	CC + TT *vs.* CT	31 (50.8%)	58 (53.2%)	1.00	0.760
		30 (49.2%)	51 (46.8%)	0.91 (0.49-1.70)	
Log-additive	CC *vs.* TT	24 (39.3%)	46 (42.2%)	1.00	0.750
		7 (11.5%)	12 (11.0%)	0.93 (0.58-1.49)	

Table S2 Comparison of *ABCB1* rs1128503 genotypes distribution in children with epilepsy with or without VPA-induced gADRs

Genetic Model	Genotype	Patients with gADRs (*n*=56)	Patients without gADRs (*n*=114)	OR (95% CI)	*p*-value
Allele contrast	C *vs.* T	40 (35.7%)	79 (34.6%)	1.00	0.850
		72 (64.3%)	149 (65.4%)	1.05 (0.65-1.68)	
Codominant	CC *vs.* CT *vs.* TT	7 (12.5%)	10 (8.8%)	1.00	0.690
		26 (46.4%)	59 (51.8%)	1.59 (0.54-4.63)	
		23 (41.1%)	45 (39.5%)	1.37 (0.46-4.07)	
Dominant	CC *vs.* CT + TT	7 (12.5%)	10 (8.8%)	1.00	0.450
		49 (87.5%)	104 (91.2%)	1.49 (0.53-4.14)	
Recessive	CC + CT *vs.* TT	33 (58.9%)	69 (60.5%)	1.00	0.840
		23 (41.1%)	45 (39.5%)	0.94 (0.49-1.80)	
Overdominant	CC + TT *vs.* CT	30 (53.6%)	55 (48.2%)	1.00	0.510
		26 (46.4%)	59 (51.8%)	1.24 (0.65-2.35)	
Log-additive	CC *vs.* TT	7 (12.5%)	10 (8.8%)	1.00	0.840
		23 (41.1%)	45 (39.5%)	1.05 (0.64-1.73)	

Table S3 Comparison of *ABCB1* rs1128503 genotypes distribution in children with epilepsy with or without VPA-induced cADRs

**Genetic Model**	**Genotype**	**Patients with cADRs (*n*=19)**	**Patients without cADRs (*n*=151)**	**OR (95% CI)**	***p*-value**
Allele contrast	C *vs.* T	18 (47.4%)	101 (33.4%)	1.00	0.090
		20 (52.6%)	201 (66.6%)	1.79 (0.91-3.54)	
Codominant	CC *vs.* CT *vs.* TT	4 (21.1%)	13 (8.6%)	1.00	0.200
		10 (52.6%)	75 (49.7%)	2.31 (0.63-8.47)	
		5 (26.3%)	63 (41.7%)	3.88 (0.91-16.43)	
Dominant	CC *vs.* CT + TT	4 (21.1%)	13 (8.6%)	1.00	0.120
		15 (78.9%)	138 (91.4%)	2.83 (0.82-9.79)	
Recessive	CC + CT *vs.* TT	14 (73.7%)	88 (58.3%)	1.00	0.190
		5 (26.3%)	63 (41.7%)	2.00 (0.69-5.85)	
Overdominant	CC + TT *vs.* CT	9 (47.4%)	76 (50.3%)	1.00	0.810
		10 (52.6%)	75 (49.7%)	0.89 (0.34-2.31)	
Log-additive	CC *vs.* TT	4 (21.1%)	13 (8.6%)	1.00	0.078
		5 (26.3%)	63 (41.7%)	1.93 (0.93-4.03)	

Table S4 Comparison of *ABCB1* rs1128503 genotypes distribution in children with epilepsy with or without VPA-induced nADRs

Genetic Model	Genotype	Patients with nADRs (*n*=48)	Patients without nADRs (*n*=122)	OR (95% CI)	*p*-value
Allele contrast	C *vs*. T	33 (34.4%)	86 (35.2%)	1.00	0.880
		63 (65.6%)	158 (64.8%)	0.96 (0.59-1.58)	
Codominant	CC *vs*. CT *vs*. TT	6 (12.5%)	11 (9.0%)	1.00	0.560
		21 (43.8%)	64 (52.5%)	1.66 (0.55-5.04)	
		21 (43.8%)	47 (38.5%)	1.22 (0.40-3.74)	
Dominant	CC *vs*. CT + TT	6 (12.5%)	11 (9.0%)	1.00	0.500
		42 (87.5%)	111 (91.0%)	1.44 (0.50-4.15)	
Recessive	CC + CT *vs*. TT	27 (56.2%)	75 (61.5%)	1.00	0.530
		21 (43.8%)	47 (38.5%)	0.81 (0.41-1.59)	
Overdominant	CC + TT *vs*. CT	27 (56.2%)	58 (47.5%)	1.00	0.310
		21 (43.8%)	64 (52.5%)	1.42 (0.72-2.78)	
Log-additive	CC *vs*. TT	6 (12.5%)	11 (9.0%)	1.00	0.870
		21 (43.8%)	47 (38.5%)	0.96 (0.57-1.62)	

Table S5 Comparison of *ABCB1* rs3789243 genotypes distribution in children with epilepsy with or without VPA-induced nADRs

Genetic Model	Genotype	Patients with nADRs (*n*=48)	Patients without nADRs (*n*=122)	OR (95% CI)	*p*-value
Allele contrast	C *vs*. T	62 (64.6%)	159 (65.2%)	1.00	0.920
		34 (35.4%)	85 (34.8%)	0.97 (0.59-1.60)	
Codominant	CC *vs*. CT *vs*. TT	19 (39.6%)	51 (41.8%)	1.00	0.930
		24 (50.0%)	57 (46.7%)	0.88 (0.43-1.80)	
		5 (10.4%)	14 (11.5%)	1.04 (0.33-3.29)	
Dominant	CC *vs*. CT + TT	19 (39.6%)	51 (41.8%)	1.00	0.790
		29 (60.4%)	71 (58.2%)	0.91 (0.46-1.80)	
Recessive	CC + CT *vs*. TT	43 (89.6%)	108 (88.5%)	1.00	0.840
		5 (10.4%)	14 (11.5%)	1.11 (0.38-3.28)	
Overdominant	CC + TT *vs*. CT	24 (50.0%)	65 (53.3%)	1.00	0.700
		24 (50.0%)	57 (46.7%)	0.88 (0.45-1.71)	
Log-additive	CC *vs*. TT	19 (39.6%)	51 (41.8%)	1.00	0.920
		5 (10.4%)	14 (11.5%)	0.97 (0.59-1.62)	
